# Supplementary material for: Modelling suggests limited change in the reproduction number from reopening Norwegian kindergartens and schools during the COVID-19 pandemic
Source: PLoS One. 2021 Feb 25;16(2):e0238268. doi: 10.1371/journal.pone.0238268 (PMC7906341; doi:10.1371/journal.pone.0238268)
Supplement: S1 File — (DOCX) [file pone.0238268.s001.docx]

**S1 File**

**Modelling Suggests Limited Change in the Reproduction Number from Reopening Norwegian Kindergartens and Schools During the COVID-19 Pandemic**

**Model parameters**

The individual based model (IBM) described in the Methods section. The parameter values used in the model simulations are presented in Supplementary Table 1.

**Supplementary Table 1**

| Parameter | Values (distribution) used |
| --- | --- |
| Transmission rate in households, $\beta_{1}$ | Varied between 0.2 and 1 per day |
| Transmission rate in schools, $\beta_{2}$ | Varied between 0.2 and 1 per day and equal to 0 before April 20, 2020 |
| Transmission rate in the rest of society, $\beta_{3}$ | Varied between 0.2 and 1 per day |
| Relative transmission rate in schools, *r* | $r=\beta_{2}/(0.94 per day)$ |
| Reduction factor for infectiousness in schools if seriously infected, $\Psi$ | 0.5 |
| relative infectiousness of individuals, $\rho_{i}$ | Chosen randomly from a Gamma distribution with mean 1 and variance 1. |
| Time dependence of infectiousness after infected, $\kappa\left( t \right)$ | Takes the form of a log-normal probability density function with parameters $\delta=-0.72$ and $\gamma=1.8$, truncated at 14 days |
| Time step in simulation, $\Delta t$ | $0.25$ days |
| Probability of serious infection ($c_{i}=1)$ | 0.1 for ages 0-9 yrs, 0.20 for ages 10-19 yrs, and 0.4 for ages >19 yrs |
| Scaling parameter for transmission as function of family size, $\alpha$ | 0.8 |
| Incubation time, $s_{i}$ | Chosen randomly for each individual from a normal distribution with a mean of 5 days and standard deviation 0.1 days. |

The probability of serious infection is highly uncertain, and we do not know the threshold for staying home from school during the pandemic. We used values that incorporate a strong age-dependence consistent with recent studies ^1,2^, and can account for pre-symptomatic and asymptomatic transmission between children. Parameters $\Psi$ and $\alpha$ are not changed from the influenza-version of the model ^3^.

**Estimation of the basic reproduction number of model runs**

The reproduction number *R* is estimated by fitting the number of infectious individuals to a simulation (without school opening) to the solution for $I(t)$ in a SEIR model:

$${\begin{aligned} {\begin{aligned} \frac{dS}{dt}=-\beta\frac{SI}{N} \\ \\ \frac{dE}{dt}=\frac{SI}{N}-a E \\ \end{aligned} \atop\frac{dI}{dt}=aE-\gamma I} \\ \end{aligned}}$$

where $S$ denotes the size of the susceptible, $E$ the exposed population, and $I$ the infected population. The *R*-estimate is obtained from $R=\beta/\gamma$, where $a=\gamma=1/(5 \mathrm{days})$, and $\beta$ is estimated from the simulation data by a numerical least-squares optimization.

**SEIR model used to estimate reproduction number from hospitalizations**

To estimate the basic reproduction number in Norway we used a one-population version of the SEIR model used by the NIPH ^4^. This model was slightly more complex than the model above, which we used to estimate $R$ from experiments of the IBM. The reason for the difference is that the time series of the number of infectious was not available, and in the spring of 2020, the $R$-estimates in Norway were based on the number of hospitalized patients. We used the same parameter values as in ^4^ , which are consistent with the ones used in the simpler SEIR model described above.

$${\begin{aligned} \frac{dS}{dt}=-\beta IS-r_{E_{2}}\beta E_{2}S-r_{I_{a}}\beta I_{a}S \\ \end{aligned} \atop\begin{aligned} \frac{dE_{1}}{dt}=-\lambda_{1}E_{1}+\beta IS+r_{E_{2}}\beta E_{2}S+r_{I_{a}}\beta I_{a}S \\ \\ \frac{dE_{2}}{dt}=-\lambda_{2}E_{2}+{\left( 1-p_{a} \right)\lambda}_{1}E_{1} \\ \\ \frac{dI_{a}}{dt}=-\gamma I_{a}+{p_{a}\lambda}_{1}E_{1} \\ \\ \frac{dI}{dt}=-\gamma I+\lambda_{2}E_{1}. \end{aligned}}$$

Here $S$ is the size of the susceptible population, $E_{1}$the exposed, $E_{2}$ the infectious but pre-symptomatic, $I_{a}$ the infectious that follow an asymptomatic course, and $I$ the infectious and symptomatic. All population variables have been normalized using the total population.

The model parameters used are $r_{E_{2}}=1.25$ and $r_{I_{a}}=0.1$ for the relative transmission rates, $\lambda_{1}=1/(3 \mathrm{days})$, $\lambda_{2}=1/(2 \mathrm{days})$, $\gamma=1/(5 \mathrm{days})$ for the characteristic time scales, and the probability of an asymptomatic course is $p_{a}=0.4$. The linearization around the disease-free fix-point at $S=S_{0}, E_{1}$= $E_{2}$= $I_{a}$=$I=0$, gives the matrix

$$A=\left( \begin{aligned} \begin{aligned} 0 \\ 0 \end{aligned} \\ 0 \\ 0 \\ 0 \end{aligned} \begin{aligned} \begin{aligned} 0 \\ -\lambda_{1} \end{aligned} \\ \lambda_{1}(1-p_{a}) \\ \lambda_{1}p_{a} \\ 0 \end{aligned} \begin{aligned} \begin{aligned} -r_{E_{2}}\beta S_{0} \\ r_{E_{2}}\beta S_{0} \end{aligned} \\ \lambda_{2} \\ 0 \\ \lambda_{2} \end{aligned} \begin{aligned} \begin{aligned} {-r_{I_{a}}\beta S_{0}} \\ r_{I_{a}}\beta S_{0} \end{aligned} \\ 0 \\ -\gamma\\ 0 \end{aligned} \begin{aligned} \begin{aligned} {-\beta S_{0}} \\ \beta S_{0} \end{aligned} \\ 0 \\ 0 \\ -\gamma\end{aligned} \right),$$

which has a trivial zero-eigenvalue and a simple eigenvalue equal to $-\gamma$. The three other eigenvalues are the roots of a cubic polynomial, and thus at least one of them is real. This eigenvalue, denoted by $\xi=\xi(\beta,\boldsymbol{c})$, which determines the stability of the disease-free fix point, depends on $\beta$ and the other model parameters (denoted by $\boldsymbol{c}$). The stability condition for the disease-free equilibrium therefore is $\xi\left( \beta,\boldsymbol{c} \right)=0$, whose solution with respect to $\beta$ is found to be

$$\beta=\frac{\gamma\lambda_{2}}{S_{0}(p_{a}r_{I_{a}}\lambda_{2}+(1-p_{a})(\lambda_{2}+\gamma r_{E_{2}}))}.$$

Hence the stability condition is equivalent to $R=1/S_{0}$, where

$$R=\beta\left( \frac{p_{a}r_{I_{a}}}{\gamma}+\left( 1-p_{a} \right)(\frac{1}{\gamma}+\frac{r_{E_{2}}}{\lambda_{2}}) \right).$$

**Estimation of R from confirmed cases**

As a supplement to the *R*-estimates obtained from fitting parameters the SEIR model to hospitalization data, we also applied EpiEstim ^5^ to the time series of confirmed infections in Norway, as well as in the cities of Oslo and Tromsø. We used a weekly window and assumed an incubation period of five days. The serial interval (time between symptom onset in pairs of infectious and infected) was assumed to be uncertain, i.e., a random probability distribution, with parameters similar to those found in ^6^: $\mu$ = 5, $\sigma_{\mu}$ = 2, $\sigma=$ 4.3, ${\sigma_{\sigma}}$= 1. Furthermore, the mean $\mu$ and standard deviation $\sigma$ was constrained to the intervals 1-9 and 3.3-5.3, respectively. The parameters in the priors (Gamma distributions) were set to the default values (mean and standard deviation equal to 5).

References

1. Davies NG, Klepac P, Liu Y, et al. Age-dependent effects in the transmission and control of COVID-19 epidemics. *Nature Medicine.* 2020;26(8):1205-1211.

2. Ludvigsson JF. Systematic review of COVID-19 in children shows milder cases and a better prognosis than adults. *Acta Paediatrica.* 2020;109(6):1088-1095.

3. Ferguson NM, Cummings DAT, Cauchemez S, et al. Strategies for containing an emerging influenza pandemic in Southeast Asia. *Nature.* 2005;437(7056):209-214.

4. Folkehelseinstituttet. Covid-19-epidemien: Kunnskap, situasjon, prognose, risiko og respons i Norge etter uke 14. <https://www.fhi.no/>. Published 2020. Accessed.

5. Cori A, Ferguson NM, Fraser C, Cauchemez S. A new framework and software to estimate time-varying reproduction numbers during epidemics. *Am J Epidemiol.* 2013;178(9):1505-1512.

6. Ganyani T, Kremer C, Chen D, et al. Estimating the generation interval for coronavirus disease (COVID-19) based on symptom onset data, March 2020. *Eurosurveillance.* 2020;25(17):2000257.

7. Cox PM, Huntingford C, Williamson MS. Emergent constraint on equilibrium climate sensitivity from global temperature variability. *Nature.* 2018;553(7688):319-322.
