# Supplementary figures and images for: Modelling suggests limited change in the reproduction number from reopening Norwegian kindergartens and schools during the COVID-19 pandemic
Source: PLoS One. 2021 Feb 25;16(2):e0238268. doi: 10.1371/journal.pone.0238268 (PMC7906341; doi:10.1371/journal.pone.0238268)

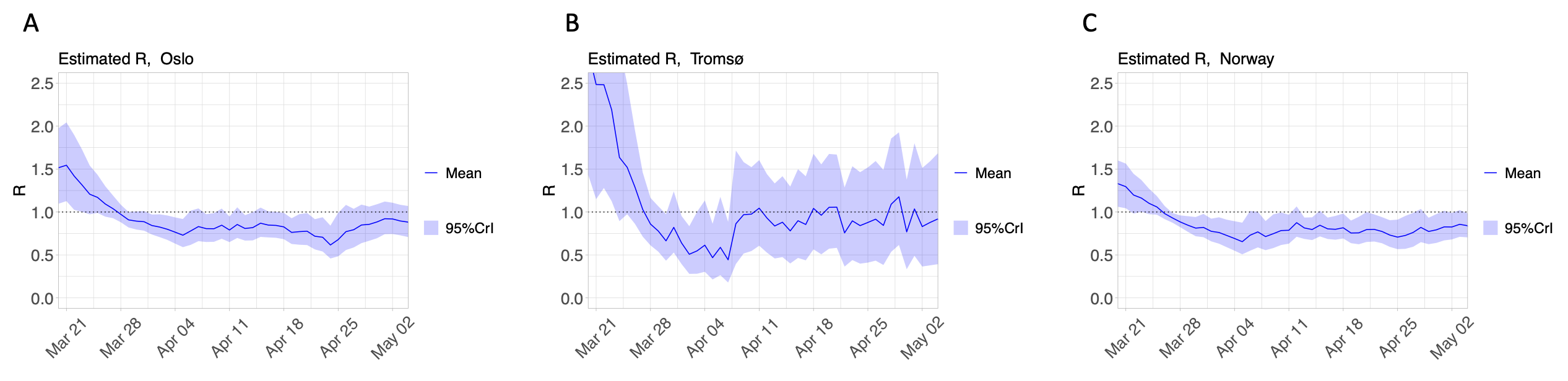

Supplement: S1 Fig — A: Estimate of R based on confirmed cases in Norway. B: Estimate of R for Oslo. C: Estimate of R in Tromsø. (TIFF) [file pone.0238268.s002.tiff]

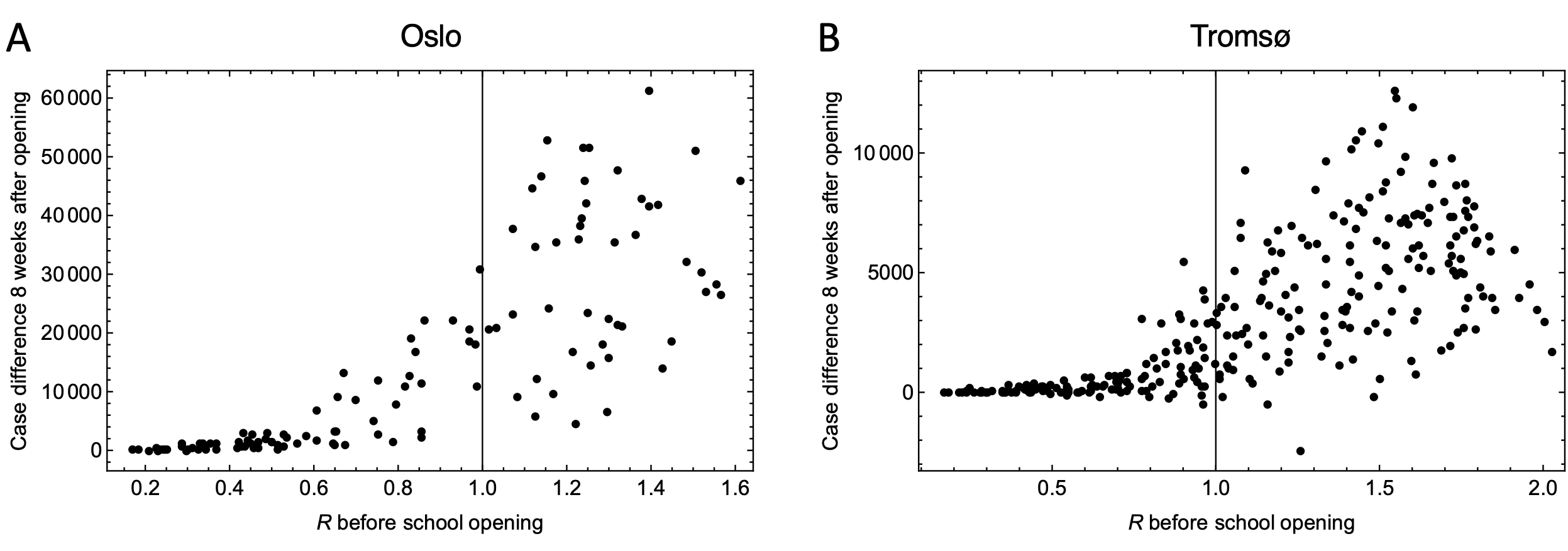

Supplement: S2 Fig — The differences in the number of cases is plotted against the R-value before school opened (April 20). Each point represents a model simulation. A: Simulations for the city of Oslo. B: The city of Tromsø. (TIFF) [file pone.0238268.s003.tiff]

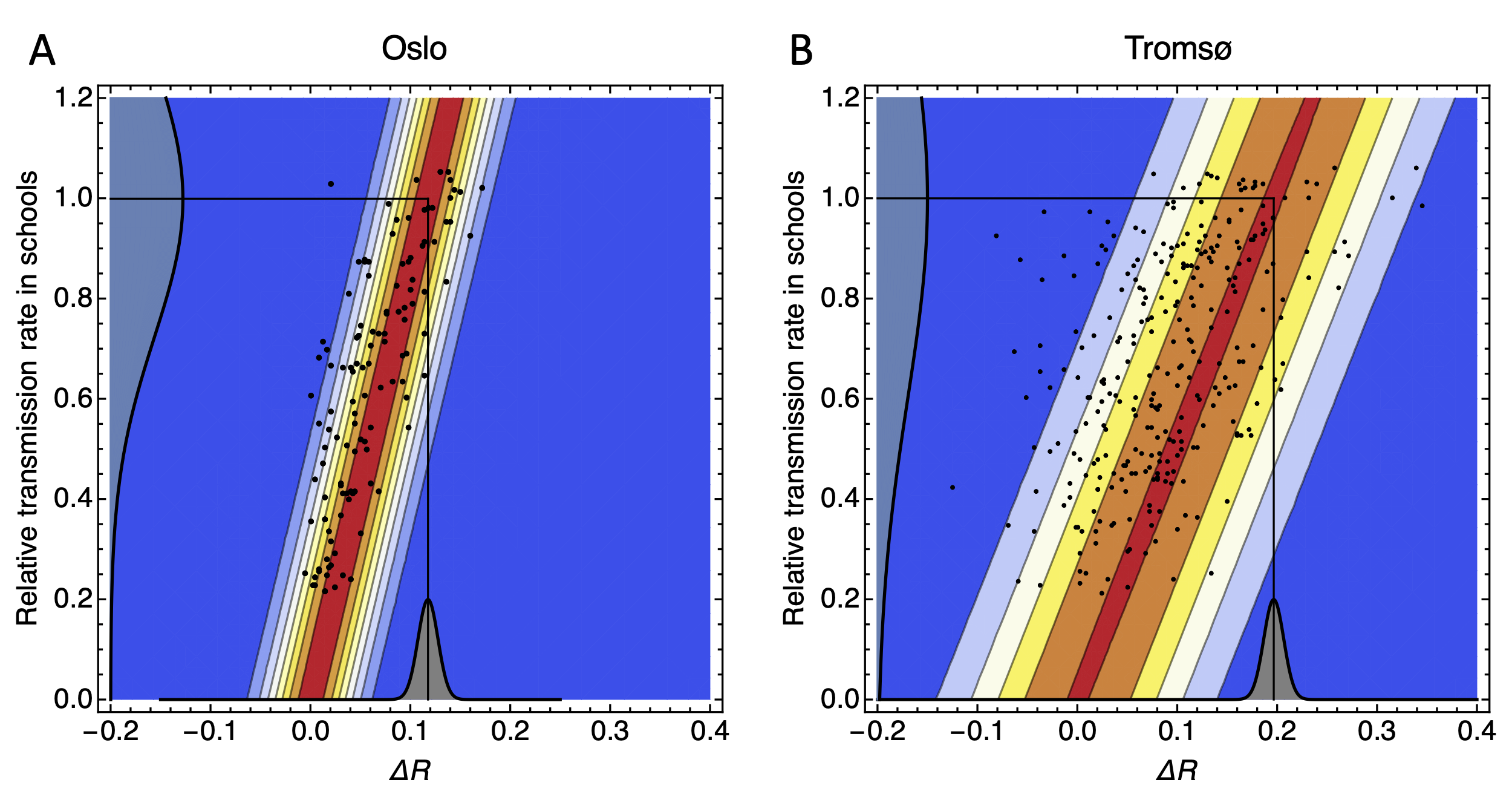

Supplement: S3 Fig — The black points are model simulations for randomly selected β-parameters, and the contours show the conditional probability density p(ΔR|r) estimated using the method in [7]. The probability densities on the axes are included to illustrate how the uncertainties are amplified. On the ΔR-axes we have chosen normal distributions p(ΔR) with standard deviation 0.02, and on the r-axes we show the corresponding distributions p(r) obtained from integration of p(ΔR|r)p(ΔR) over ΔR. A: For the city of Oslo. B: For the city of Tromsø. (TIFF) [file pone.0238268.s004.tiff]
