# Supplementary material for: Modelling suggests limited change in the reproduction number from reopening Norwegian kindergartens and schools during the COVID-19 pandemic
Source: PLoS One. 2021 Feb 25;16(2):e0238268. doi: 10.1371/journal.pone.0238268 (PMC7906341; doi:10.1371/journal.pone.0238268)
Supplement: Attachment — Submitted filename: Reviewers_comments.docx [file pone.0238268.s005.docx]

**Re: Modelling suggests limited change in the reproduction number from re-opening Norwegian Kindergartens and schools during COVID-19**

In the manuscripts, the authors considered the effect of kindergartens and schools opening on COVID-19 dynamics in Norway. Both basic and effective reproduction numbers were estimated relative schools openings via the individual-based model (IBM). The found little relative change in reproduction number from opening the schools and concluded that controlled school opening might not alter the cause of progress made on COVID-19 in many countries considering this option. The methodology and results are well-written and supported by good discussion and conclusion. In general, the manuscript is well-organized, and my comments on the work are below:

Major comments:

1. The authors follow the stochastic model for influenza directly in [1] for COVID-19. The dynamics of COVID-19 is entirely differently from influenza [2]. I can see that some of the parameters are updated for COVID-19, but pre-symptomatic transmission and asymptomatic are two of the factors that make COVID-19 unique. These are not accounted for in this modelling. Thus, I wonder if this stochastic simulation captures the essential dynamics of COVID-19.
2. It is has been shown that there exist age-dependent susceptibility and infectivity in COVID-19 dynamics [3]. More of this assertion comes from a population with more younger age group than older age group vs with a population with more older age group than younger age group. The Norway age-structure is pyramid [4], meaning there are fewer young children than adults. Thus, allowing contacts between children and adults via school re-opening may not significantly impact COVID-19. However, all these need to be adjusted for in the modelling. How does infectiousness and susceptibility of younger children differ from adults in this modelling? Note, your $c_{j} and \rho_{j}$ may answer relative infectiousness but not relative susceptibility.
3. As mention in 1 above, COVID-19 has its dynamics. Thus, a SIR or SEIR compartmental like models may be over-simplification of reality. The authors did not provide the compartmental like nature of their IBM. Hence, it is easier to assume it is SIR. Also, what is the essence of using SEIR model to estimate $R_{0}$? The IBM model can be fitted to data to estimate this. How does the dynamics of your SEEIIR model differ from IBM. Since Age is considered, I guessed age-structured compartmental model with appropriate contact matrix would be closed the approximation of the IBM model.
4. The results are a little bit difficult to follow. For instance, “ For each city, the figures are based on the whole ensemble of simulations but weighted by a probability density $p(R)$ for the reproduction number prior to April 20,…”. $p(R)$ is not defined anywhere else, even in the supplementary! I may miss this.
5. Similar to 4 above, “ the Gaussian weight distribution is centred around R=0.6, 0.8 and 1.0, …”. Which Gaussian distribution? All you have in figure 2 are straight lines of the basic reproduction numbers. “In panel B, the probability of having $R+\Delta R>1$ after school opening in Oslo is estimated be 0.13…”. How? Nothing in the methodology pointed to this, and even in panel E, figure 2, what you have are straight lines. Maybe a better figure that depicts these changes will be better.

Minor comments:

1. Change “serios” to “serious”
2. “Form” to “are” in the model runs and analyses section

References:

1. Ferguson, N. M., Cummings, D. A., Cauchemez, S., Fraser, C., Riley, S., Meeyai, A., ... & Burke, D. S. (2005). Strategies for containing an emerging influenza pandemic in Southeast Asia. *Nature*, *437*(7056), 209-214.
2. <https://www.who.int/news-room/q-a-detail/coronavirus-disease-covid-19-similarities-and-differences-with-influenza>
3. Davies, N. G., Klepac, P., Liu, Y., Prem, K., Jit, M., Eggo, R. M., & CMMID COVID-19 working group. (2020). Age-dependent effects in the transmission and control of COVID-19 epidemics. *MedRxiv*.
4. https://www.populationpyramid.net/norway/2019/
